# Development and feasibility of a telemedicine tool for patients with recurrent urinary tract infection: myRUTIcoach

**DOI:** 10.1007/s00192-023-05634-x

**Published:** 2023-09-27

**Authors:** J. J. Pat, C. C. E. T Pape, M. G. Steffens, L. P. W. Witte, M. H. Blanker

**Affiliations:** 1 Department of General Practice and elderly Care Medicine, University of Groningen, University Medical Centre, Groningen, Hanzeplein 1, 9713 GZ Groningen, The Netherlands; 2 Department of Urology, ISALA Clinics, Dokter van Heesweg 2, 8025 AB Zwolle, The Netherlands; 3 Erasmus School of Health Policy & Management, Erasmus University Rotterdam, Burgemeester Oudlaan 50, 3062 PA Rotterdam, The Netherlands

**Keywords:** eHealth, RUTI, Qualitative research, NASSS

## Abstract

**Introduction and hypothesis:**

Patients with recurrent urinary tract infection (rUTI) have limited knowledge of preventive strategies to lower the risk of UTI. We aimed to develop and test the feasibility of an eHealth system for women with rUTI, named myRUTIcoach, and explored the facilitators and barriers related to its adoption.

**Methods:**

We developed myRUTIcoach in a structured iterative process and tested its feasibility among 25 women with rUTI over 2 months. Subsequent questionnaires covered satisfaction, accessibility, and experiences with myRUTIcoach. A random selection of participants and relevant stakeholders took part in semi-structured interviews to explore adoption. Data were analyzed and elaborated using inductive and deductive approaches using the Non-adoption, Abandonment, Spread, Scale-up, and Sustainability (NASSS) framework.

**Results:**

MyRUTIcoach was not only widely accepted but also facilitated communication with health care professionals (HCPs) and contributed to greater knowledge of rUTI. Women graded the system a mean of 8.0 (±0.6) out of 10, with 89% stating that they would recommend it to others. Patients indicated that self-management skills were the major facilitators and barriers related to adoption, whereas HCPs stated that the disconnect between myRUTIcoach and electronic health care records (EHRs) was the major barrier.

**Conclusions:**

This research describes the development and testing of myRUTIcoach for women with rUTI. Patients and HCPs reported high satisfaction and compliance with myRUTIcoach. However, adoption by the intended users is complex and influenced by all examined domains of the NASSS framework. We have already improved linkage to EHRs, but further optimization to meet patient needs may improve the effectiveness of this self-management tool for rUTI.

**Supplementary information:**

The online version contains supplementary material available at 10.1007/s00192-023-05634-x

## Introduction

Urinary tract infections (UTIs) are among the most frequent bacterial infections worldwide [1]. Most occur in otherwise healthy nonpregnant women with no structural or functional abnormalities of the genitourinary tract. However, despite being uncommon for most women, some can experience recurrent UTIs (rUTIs). The therapeutic approach in this group focuses on prevention and self-management [2], with typical strategies including behavioral modification and both non-antimicrobial and antimicrobial prophylaxis [3]. A recent qualitative study among women with rUTI indicated marked variation in their knowledge of preventive measures [4].

Patient education is the foundation of both personalized care plans and compliance with preventive measures [5], with eHealth increasingly coming to the fore as an approach to improve both education and prevention. Indeed, eHealth approaches have been implemented successfully for several chronic conditions, including hypertension, inflammatory bowel disease, and diabetes mellitus [6]. Complementing this practice shift, evidence shows that patient empowerment, direct involvement by health care professionals (HCPs), and integrated care, improve chronic disease outcomes [7]. Nevertheless, eHealth technologies are often used incorrectly by the intended end-users, leading to failed implementation in health care systems [8].

Adoption is a prerequisite for the successful implementation of eHealth, requiring patients to integrate the technology into their daily routines. Although several self-management apps exist for use by patients with UTIs, they suffer from both very low concordance with current guidelines (e.g., lack of treatment information) and insufficient evidence of their effectiveness in reducing UTIs. Limited HCP involvement during app development, coupled with an inability to guide behavioral changes interactively, could also explain their ineffectiveness.

We aimed to develop an eHealth app, for use by women with rUTI, that accurately reflected the latest international guidelines, before studying its feasibility in terms of end-user compliance, satisfaction, and adoption.

## Materials and methods

### Development of myRUTIcoach

We developed an online app-based intervention called myRUTIcoach, comparable with the previously studied myIBDcoach [6]. This included its overall design, content development, and technical development.

The content of myRUTIcoach was designed by a development team comprising two urologists, a PhD student, and a general practitioner (GP). Patient input came from an earlier qualitative study [9]. The development team discussed the design of the telemedicine program, the relevant topics, and eligible questionnaires. Topics for the e-learning modules were selected and developed by the development team, and to ensure readability, were checked by an independent writer for comprehension by a broad target audience. All information in the e-learning modules was developed using international guidelines [2, 10]. The e-learning modules also included explanatory videos.

Sananet BV (Sittard, the Netherlands), a company that specializes in the development and implementation of telemedicine and self-management tools, performed the technical development. They not only integrated relevant information and questionnaires into both a web-based and an HTML version of the myRUTIcoach app but also created secure links between patients and treating hospitals. The app included modules for monitoring, outpatient visits, e-learning, and personal care plans, together with an administrator module for use by HCPs. The personal care plan and administrator modules are standard for all coaching software produced by Sananet, and as such, were not developed specifically for the current app.

The myRUTIcoach app meets all requirements of European law regarding the security and confidentiality of patient data.

Our study was approved by our local medical ethics committee.

### Feasibility study of myRUTIcoach

After developing the app, we conducted a feasibility study to evaluate patient compliance, satisfaction, accessibility, and experience with myRUTIcoach among 25 consecutive women recruited from our clinical center between December 2020 and February 2021. Adult women were eligible for inclusion if they met the criteria for rUTI (>3 UTI/year or >2 UTI/6 months) [2]. They were excluded if they met any of the following criteria: urinary catheter use, neurogenic bladder dysfunction, previous urogynecological surgery, previous urological malignancy, no internet access, and the inability to read and understand the Dutch language. After signing informed consent forms, participants received unique login details for the app (username and password). During the 2-month study period, patients were asked to complete all e-learning modules and a monitoring module twice a month. After the study period, patients were then asked to complete an evaluation questionnaire covering satisfaction, accessibility, and experiences with myRUTIcoach.

### Facilitators and barriers related to adoption

**Fig. 1:**
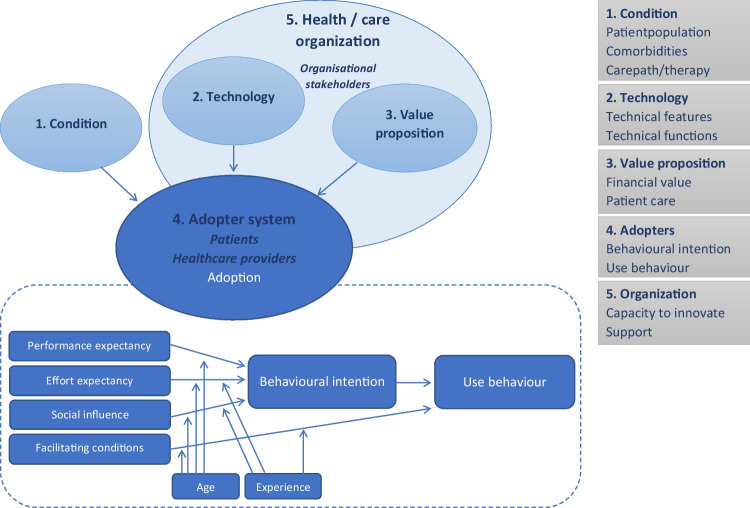
Composed framework combining the Non-adoption, Abandonment, Spread, Scale-up, and Sustainability (*NASSS*) framework and the Theory of Acceptance and Use of Technology (*UTAUT*) model. The UTAUT model was added to domain 4 of the NASSS framework to explore end-user adoption of myRUTIcoach

Understanding the interaction between individual adoption (micro-level) and the organizational context (meso-level) can explain and predict why an implementation is successful or not in a given organization [8]. We qualitatively investigated the facilitators and barriers related to myRUTIcoach adoption by adapting the Non-adoption, Abandonment, Spread, Scale-up, and Sustainability (NASSS) framework to our research (Fig. 1). This framework seeks to understand and explain why technological innovation programs in health care fail [8]. Specifically, we examined five domains of the NASSS framework: condition, technology, value proposition, adopter system, and health/care organization. The analysis emphasized domain 4, and to better describe user intent and behavior, we added the “Unified Theory of Acceptance and Use of Technology” (UTAUT) model to this domain [11]. Questions were based on the NASSS Complexity Assessment Tools for interviews [12]. This helped to determine complexity (simple, complicated, or complex) for each domain, which is known to affect adoption [8]. Adoption is known to decrease as the complexity of a technology or organization increases.

Individual adoption by end-users (patients and HCPs) was explored by semi-structured interviews. We recruited newly registered patients from the outpatient clinic and feasibility study and divided them into three groups: patients who completed the pilot study in full; patients who completed the pilot study, but who did not complete the final evaluation; and patients who stopped during the pilot study and did not complete the final evaluation.

To provide insight into the organizational context, we also involved various stakeholders from ISALA (a PhD student, project manager, data analyst, and financial controller). Finally, we asked questions from domains relevant to the intended user or stakeholder. Appendix 1 includes the theoretical background.

### Data collection and outcome measures

Patients provided demographic information at recruitment. Feasibility data (i.e., satisfaction, accessibility, and experiences) for the myRUTIcoach app were collected from patient evaluation questionnaires and qualitative interviews with a random selection of patients. During the development and implementation phases, we interviewed all participants regarding the facilitators and barriers affecting adoption. For the interviews, we invited all participants of the feasibility study by letter and all newly referred patients by telephone before their first appointment. The patient interviews continued until saturation. Concerning stakeholder involvement, four HCPs who were going to work with the myRUTIcoach were invited. The development team included two urologists. We interviewed four organizational stakeholders, either in-person or digitally, with interviews lasting 5 to 60 min.

### Statistical analyses

Patient demographic and feasibility data were analyzed descriptively using IBM SPSS for Macintosh, version 27.0 (IBM Corp., Armonk, New York, USA). Qualitative interviews were recorded and transcribed verbatim for analysis by two researchers (LP and MDM) in Atlas.ti version 9. The analytical process was done by combining inductive analysis (data driven) and deductive analysis (theory driven; based on the NASSS framework and the UTAUT model). Coding was done in initial and targeted selective phases. After refining the coding scheme, correlations between categories were explored to raise the level of analysis from categorical to thematic, aiming to interpret the data in a meaningful way. For each domain, the level of complexity (simple, complicated, or complex) was determined using the criteria detailed in Appendix 1.

### Ethical considerations

The study was approved by the Medical Research Ethics Committee of ISALA, under trial number 201026.

## Results

### Development of myRUTIcoach

**Fig. 2:**
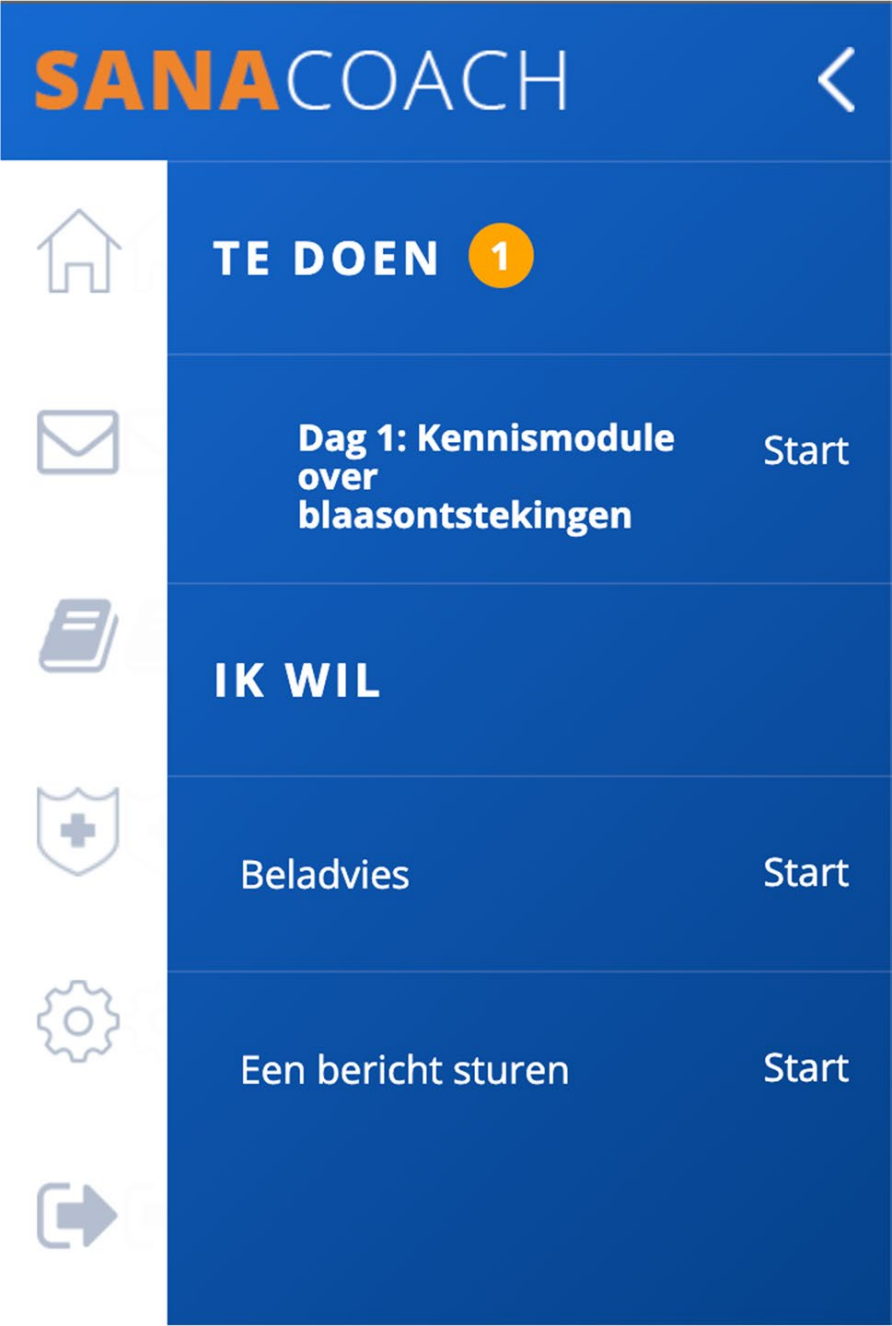
Screenshot of the homepage of myRUTIcoach. From this page patients can start e-learning, open their personal care plan, or send a message to their health care professional

**Fig. 3:**
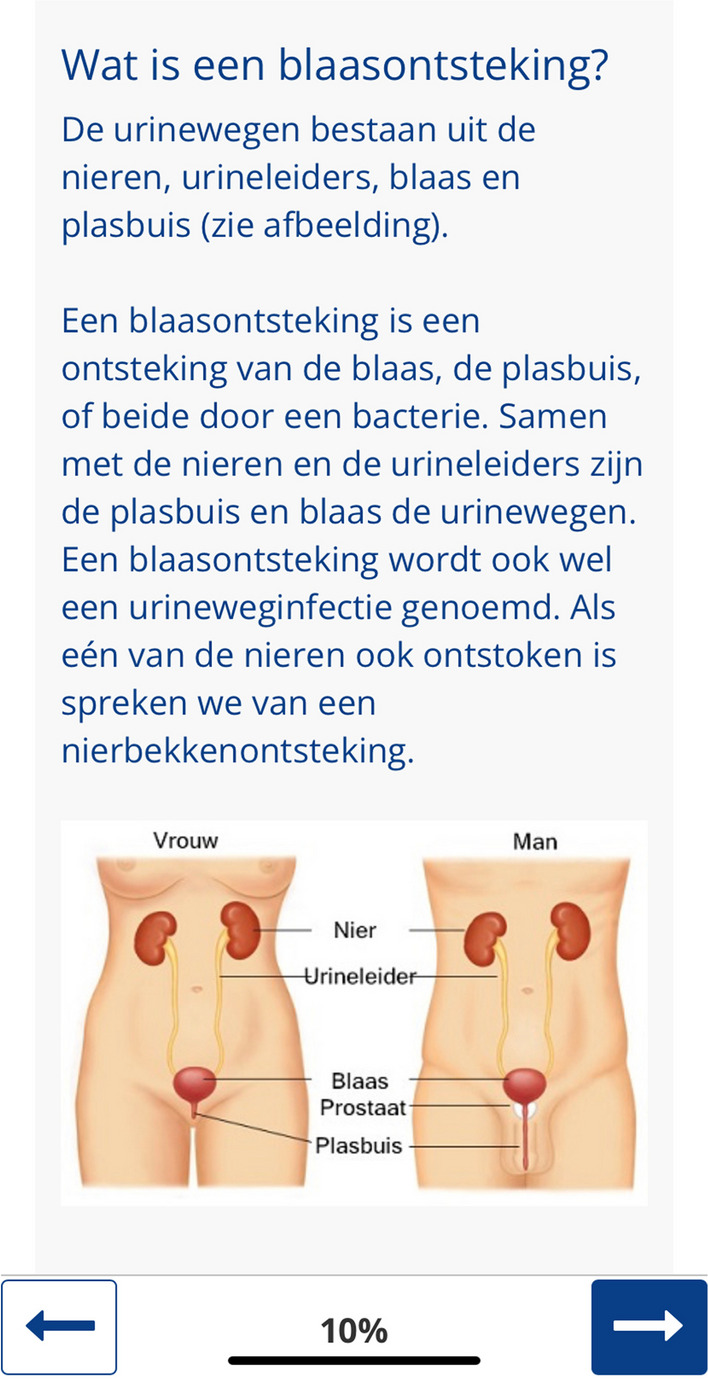
Screenshot of an e-learning module. Patients can navigate using the arrows at the bottom. The written information is also visualized using images or videos

The myRUTIcoach app comprises modules for e-learning, monitoring, personal care plans, and administration. All technical information and modules are summarized in Table 1. Figure 2 is a screenshot of the homepage of myRUTIcoach. From this page patients can start e-learning, open their personal care plan, or send a message to their HCP. Figure 3 is a screenshot of an e-learning module. Patients can navigate using the arrows. The information is also visualized using images or videos.

**Table 1:** Summary of technical information and modules

Section	Function	Frequency
Monitoring module	Monitors patient-reported disease activity, patient-reported risk factors (fluid intake, hygiene, coitus), antibiotic usage, antibiotic prescriber, quality of life (HowRu questionnaire), and patient knowledge of recurrent UTI	Twice a month
E-learning module	Offers patient-tailored information on six selected topics (definition of UTI, anatomy and function of the urinary tract, risk factors for rUTI, how to recognize a UTI, what to do in case of an acute UTI, and self-management of rUTI)	First week of use. Restarted when applicable
Communication	Facilitates communication between patient and HCP and provides systematic documentation of communication	Available 24/7
Personal care plan	Gives a clear overview of follow-up for both patient and HCP	Available 24/7

The developer was Sananet BV, and the app was designed to be operable on three systems (iOS, Android, and HTML)

*rUTI* recurrent urinary tract infection, *HCP* health care professional

#### E-learning modules

The interactive e-learning module included the following topics: “Definition of UTI,” “Anatomy and function of the urinary tract,” “Risk factors for rUTI,” “How to recognize a UTI,” “What to do in case of an acute UTI,” and “Self-management of rUTI” (see Appendix 2 for specific contents). Each topic started automatically in the first week of use, and when appropriate, patients and HCPs could choose to restart an e-learning topic.

#### Monitoring modules

For monitoring, we recorded data on patient age, rUTI duration, smoking status, family history, sexual activity, and lifestyles relevant to UTIs (e.g., fluid intake, cranberry supplements, genital soap use, condom use, post-coital voiding, and taking sufficient time to void). Participants recorded these topics in the app as part of a baseline assessment. Lifestyles were scored on five-point Likert scales ranging from never to always.

Patients were requested to complete a regular monitoring module twice monthly. This included questions on disease activity, medication use, the prescriber, satisfaction, and general quality of life (modified HowRu questionnaire) [13]. The values recorded from these questionnaires were then presented in a clear overview for use by the HCP. At the end of the monitoring module, patients were asked a random selection of 3–4 of 13 questions developed for the e-learning modules. If answered incorrectly, the questions were asked again in the following monitoring module. If the patient continued to answer incorrectly, they were asked to repeat the relevant e-learning modules.

#### Personal care plan

The personal care plan detailed all follow-up, prevention, and treatment information discussed during outpatient visits.

#### Administration page

The administrator page provides an overview of all participating patients for use by the patient’s HCP (i.e., urologist or urology resident). To ensure the safety of home monitoring, alerts (red flags) were created in this module when values recorded from questions in the monitoring module exceed predefined thresholds (e.g., hematuria, fever >38.5°C, flank pain). When an alert was generated, patients were advised to contact their HCP for further evaluation. Based on the extent and severity of a given complaint, the HCP could then decide whether the patient should be seen at the outpatient clinic.

Patients could communicate easily with their HCP by sending a message to the administrator page through a secured connection. Every new message created an alert in this module.

### Results of the feasibility study

The myRUTIcoach app was piloted among 25 patients with rUTI. Table 2 details the patient characteristics. During the 2-month study period, 15 (60%) contacted their HCP at least once through myRUTIcoach. Most questions were related to mild symptoms of a possible UTI (*n*=15) or to queries about medication (*n*=6). Of these, 13 (86%) reported that the digital communication positively affected their contact with the HCP.

**Table 2:** Baseline characteristics of participants in the feasibility study

Characteristic	Participants (*n*=25)
Age, mean (SD)	57.9 (19.0)
Postmenopausal, *n* (%)	13 (52%)
Disease duration (years), mean (SD)	3.2 (0.7)
UTI frequency (mean number of UTI/year)	5.6 (range 3–18)
Lifestyle	3.59

*SD* standard deviation, *UTI* urinary tract infection

All but 2 patients (92%) completed all e-learning modules and the final evaluation (1 completed half of the modules and the other completed none). After completion, most patients felt that the modules had improved their knowledge of rUTI. Patients scored the myRUTIcoach 8 out of 10 overall. Patients rated the accessibility of the system highly, and 22 patients (89%) stated they would recommend myRUTIcoach to other patients. Overall lifestyle scores improved from 3.59 on the five-point Likert scale to 3.82 at 1 month and 3.83 at 2 months.

### Facilitators and barriers related to adoption

We conducted 25 semi-structured interviews with 17 patients (13 from the pilot study and 4 new participants), 4 health care providers, and 4 organizational stakeholders. The pilot study participants were divided into groups of 7 (group 1), 4 (group 2), and 2 (group 3). The following results were then discussed by NASSS domain, first addressing the complexity and then the rationale through the different perspectives and associated facilitators and barriers. However, the interdependence between domains remained and this approach was only used to provide structure. By exploring the interactions between technology adoption and the organizational context in different domains, we sought to clarify the factors influencing myRUTIcoach adoption. Table 3 shows a summary of the results.

**Table 3:** Summary of themes, facilitators, barriers, and complexity of Non-adoption, Abandonment, Spread, Scale-up, and Sustainability (NASSS) domains

Domain and complexity	Theme	Facilitator	Barrier	Facilitator and barrier
Condition, complicated	Nature of disease		Heterogeneous population	Complexity of therapy
	Care path/therapy			
	Other factors		Range of age	Self-management
Technology, complex	Technical functions	Simple preparation	Linkage with EHR	
	Technical features	Support present	Indirect measurements	
Value proposition, complicated	Financial value	Business case well-defined	Financial value negative	
	Patient care	Added value (P+HCP)	Unknown effectiveness and cost-effectiveness	
Adopters, complicated	Adoption/use	User-friendliness (P)	Deficient digital skills (P)	User-friendliness (HCP)
		Added value (P+HCP)		
		Support from family/carer (P)		
		Work routine (HCP)	Division of labor not final	
	Intention	Digital competence (HCP)	No value (P)	Experiencing no symptoms
Organization, simple	Capacity to innovate	Good management structure and relationship		
		Great organization with proper resources and human resources		
		Good organization system fit		
	Support	Open for innovations		

*EHR* electronic health record, *HCP* health care professional, *P* patient

#### Domain 1: condition

Three main factors complicated the condition domain for rUTIs. First, the patient group was heterogeneous owing to the variety of symptom presentations, perceptions of the condition, and age at presentation. HCPs therefore considered treatment with preventive measures to be person specific and difficult to predict at this level. Second, patients had divergent knowledge and skills about self-management, meaning that experiences with myRUTIcoach varied. Third, the current care pathway is not sufficiently efficient or effective, which encouraged divergence or variation.

Clinician: “That cycle in which you actually have three parties who are dissatisfied: a GP who can't solve the problem, a patient who has a recurring problem, and specialists who get the referral, but can't actually do very much extra either, except for diagnostics that don't yield anything, and then repeating all the advice one more time. That's just kind of a stalemate that leads to high care demand and low satisfaction for all parties.”

#### Domain 2: technology

The technology used for myRUTIcoach was deemed complex owing to the disconnect between the app and the patient’s electronic health record (EHR), which reduced user friendliness for HCPs. Also, the data analyst suggested that this technical link might be crucial for monitoring results and investigating whether set goals have been achieved.

#### Domain 3: value proposition

The value proposition was complicated by the unknown effectiveness, cost-efficiency, and negative financial value for the organization. Currently, expectations only exist for the effectiveness and cost efficiency of the new care pathway, with the business case made by the financial controller predicting that introducing myRUTIcoach would have a negative financial impact for the organization. This was based on the expectation of fewer patients being treated at the outpatient clinic, thereby reducing costs and revenues. Overall, these factors complicate this domain. The well-defined business case and positive added value for patients and providers are explained further in domain 4.

#### Domain 4: adopter system

This domain is complicated because other factors influencing adoption, with the specific facilitators and barriers differing among the intended users.

Facilitators of adoption among patients include awareness of self-management, accessibility, user friendliness, help from loved ones, and the (expected) added value. Most patients experience added value through information and education, the advice function, contact with their HCP, and the feeling of support.

Patient: “People are watching every now and then, and that was a pretty reassuring thought for me.”

Barriers to adoption among patients include poor digital skills and not expecting or experiencing added value. The myRUTIcoach app did not add value for patients who experienced care from their GP as sufficient, who did not need extra care, who already knew and applied the information provided, and who experienced the repeated questionnaire completion as meaningless.

Patient: “But at a certain point, I was like, ‘Yes, it repeats all these questions and the answers too.’ [...] But yes, the situation doesn't actually change at that point. [...] and I also found that the same questions came back very often. Yes, I did all that already and it doesn't change anything.”

Factors considered to both facilitate and hinder adoption were symptom resolution and self-management skills. Symptom resolution could facilitate app use when patients felt that it helped to manage their symptoms, but it could also act as a barrier when patients felt that they had reached their goal and no longer needed to use the app. Awareness of self-management skills also influenced therapy adherence and myRUTIcoach adoption. Patients who consciously used myRUTIcoach to promote self-management continued to use the app.

Patient: “Well because then you actually have a tool at hand to address the problem […]. The app asks questions and completing them every time is kind of a learning process.”

By contrast, patients who lacked awareness of self-management skills seemed to use the app for low-threshold contact with an HCP or even stopped using it entirely.

Patient: “Yes it did help me, but I wouldn't want him all the time. [...] Maybe a pat on the back once a month [...] So if you do have cystitis every time … I think I'll think about it differently then. But then maybe there are other things going on. Then maybe there is something with the bladder after all.”

Facilitators mentioned by HCPs included the potential added value to both the care pathway and patient care, fitting myRUTIcoach into work routines, and having the requisite digital skills. Perceived barriers were the lack of a clear division of labor and the lack of linkage between myRUTIcoach and the EHR. Workability and usability appeared to depend on system linkage.

#### Domain 5: health/care organization

This domain is quite simple owing to the innovative nature of our organization, the positive and supportive attitude of organizational stakeholders, the support provided by the Connected Care Centre (CCC), and the regular reviews and meetings for the development and implementation of myRUTIcoach. According to the project leader, the driving force of the urology department (physician–researcher) was an important stimulant for developing the app and had a positive influence on acceptance of the new technology among HCPs. However, the undefined role of the CCC and support staff did increase the complexity of using and implementing the myRUTIcoach app.

## Discussion

This feasibility study has shown that the newly developed myRUTIcoach is acceptable to patients and HCPs as a self-management tool for women with rUTI. In particular, patients indicated that the tool facilitated communication with HCPs and improved their knowledge of rUTI. Qualitative evaluation revealed that several factors influenced the adoption of myRUTIcoach by the intended users and increased the intervention’s complexity. The Dutch National Health Care Institute (*Zorginstituut Nederland*) has recently stated that significant room exists to improve the treatment of UTI [14]. This report identified three pillars: to improve shared decision making, to reduce antibiotic prescribing, and to reduce the use of diagnostics [14]. Each of these is targeted by the myRUTIcoach app.

To increase patient empowerment, myRUTIcoach contains e-learning modules, promotes patient self-management, and facilitates communication between patients and HCPs. The myRUTIcoach app benefits from being accessible on a computer, smartphone, or tablet. Moreover, the majority of women (90%) would recommend myRUTIcoach to other patients, reflecting a high degree of patient satisfaction. Similar satisfaction rates were found in the feasibility study for myIBDcoach, which has a comparable purpose for patients with inflammatory bowel disease. When using myIBDcoach, patients required significantly fewer outpatient visits and hospital admissions, while also reporting a high quality of care [6]. The (cost) effectiveness of the myRUTIcoach has yet to be established.

The current pilot study has provided valuable insights into the potential impact of myRUTIcoach. Of note, positive changes in preventive behavior were observed as early as 4 weeks. Although further improvement was possible, these changes stabilized during the last 4 weeks. To the best of our knowledge, no other research has shown that interactive eHealth-based education for UTI could positively influence preventive behavior. Most self-management apps for the treatment of UTIs appear to lack effectiveness, at least in part, because they were not developed specifically for rUTI and they have limited ability to direct behavior change [15]. By seeking input from intended users to optimize myRUTIcoach adoption may have helped to produce a more effective self-management tool.

Despite the proven benefits of telemedicine tools for chronic conditions, to date, no such tool has been developed for rUTI. A possible reason may be that the affected population is heterogeneous and has many elderly patients who lack basic digital skills, which may hinder adoption. This is also described in a scoping review on the barriers and facilitators related to eHealth use among older adults [16]. Sananet provides a detailed handbook for all modules in our telemedicine tool, with further support available to all users via a helpdesk.

Adoption of the myRUTIcoach can be optimized by understanding the relevant barriers and reducing them whenever possible. For example, we received feedback that unclear division of tasks and lack of connection with the EHR were important barriers to adoption. Since completing the current research, we have connected the myRUTIcoach app to the EHR and have clarified the division of labor between HCPs and support staff. This has included further training for both support staff and HCPs. By influencing the complexity of the affected domains, these changes should positively increase adoption.

We could find no other research that has systematically developed a telemedicine app for use by women with rUTI. The development of myRUTIcoach relied on input from relevant experts and stakeholders in this field. However, we did not include patients in the development process for the current research because patient preferences had already been ascertained in an earlier qualitative study [9]. This study is also one of the first to use the NASSS Complexity Assessment Tools for interviews, although it should be noted that this approach can only generate unstructured data [12]. Nevertheless, the instrument afforded us the ability to explore the complexity of our innovation and to adapt flexibly to the research.

## Conclusion

This feasibility study of the newly developed myRUTIcoach app revealed high patient adherence and satisfaction. All examined domains of the NASSS framework appeared to influence the adoption of myRUTIcoach by the intended users. Identified barriers can be tackled in both future iterations of the app and during their implementation. The ability of myRUTIcoach to generate lifestyle change makes it a potentially effective self-management tool for the treatment of rUTI.

### Supplementary information

ESM 1 (DOCX 1033 kb)

ESM 2 (DOCX 33 kb)

## Abbreviations

CCC: Connected Care Centre
EHR: Electronic Healthcare Record
GP: General Practitioner
NASSS: Non-adoption, Abandonment, Spread, Scale-up, and Sustainability
UTUAT: Unified Theory of Acceptance and Use of Technology
UTI: Urinary tract infection
HCP: Health care professional
QoL: Quality of life
RUTI: Recurrent urinary tract infection
ZIN: Zorginstituut Nederland
