# Supplementary material for: Development and feasibility of a telemedicine tool for patients with recurrent urinary tract infection: myRUTIcoach
Source: Int Urogynecol J. 2023 Sep 27;34(11):2817–25. doi: 10.1007/s00192-023-05634-x (PMC10682280; doi:10.1007/s00192-023-05634-x)
Supplement: Supplementary file 1 — (DOCX 1033 kb) [file 192_2023_5634_MOESM1_ESM.docx]

# Appendix 1: Method

Table of contents:

1. Theoretical background
2. Relevant domains per end-user or organisational stakeholder
3. NASSS framework for complexity
4. References

*Author: C.C.E.T. Pape*

*Date: 11-12-2022*

## 1) Theoretical background

In this section, the concept of adoption is defined and explained. Next, the UTAUT model and the NASSS framework will be explained and the composed framework for this study will be described.

### 1.1 Adoption

Adoption in this study refers to integrating the technology into the individual's daily practice. Before adoption occurs, the technology must first be accepted (acceptance), after which a person has the intention to use the technology (behavioral intention) and uses the technology (usage behavior). Acceptance and adoption are related to the concept of "relative advantage," which is defined as "the degree to which the potential end user believes that the innovation is better than what it was beforehand" (1). This is part of the Innovation and Diffusion Theory (IDT), where the decision to approve or reject an innovation is influenced by five innovation characteristics: observability, relative advantage, compatibility, testability, and complexity. The first four characteristics are positively related to the rate of adoption, while complexity is inversely related to adoption (1).

In addition, the concept of "adherence" plays an important role in the adoption of a technology. Adherence refers to whether a technology is used as it is intended by the developers (2). When patients adhere to the therapy provided from the technology, the effectiveness of the technology can be examined. Many technologies will be developed in such way that the more it is used, the more effective it will be. However, people may stop using a technology because it has been sufficient and therefore it is no longer necessary to use it (2).

Research on eHealth adoption is often done separately at the micro- and meso-levels (3), while literature shows that factors at these levels are interdependent (4). A study of eHealth adoption in Dutch hospitals examined factors, which influence adoption at the organizational level (3). The adoption of eHealth seems to stabilise in the phase of interest and "commitment" (dedication), which is significantly determined by three factors. These factors are: hospital size, top management support, and organizational readiness (3). Here, organizational readiness is a partial mediator for hospital size and support from top management. However, this study lacks a description of adoption at the micro-level. The study by Nieboer et al showed that the perceptions and values of Dutch healthcare professionals (partly) determine the success of an implementation of a technology (5). Factors for a successful implementation were the reliability of the technology, training of team members in the practical use of the new technology and the availability of a help desk. Barriers to adoption by healthcare professionals include lack of user-friendliness, fear of changes to their work routine, job loss, or patient safety and well-being. A study of patient adoption of eHealth describes that adoption depends on individual facilitating and hindering factors, which are unique in each situation (6). Adoption by patients is most influenced by accessibility of the technology and user-friendliness 6). Studies on urologic patients' adoption of self-management applications are not yet known.

For the adoption of a technology, it is important to involve all stakeholders of an eHealth technology (7). Often, technology developers and healthcare professionals design eHealth technologies for the goals they have identified. To a lesser extent, the context and personal needs and desires of end users are taken into account (2).

### 1.2 UTAUT

After a technology is accepted, a person can have an intention to use the technology (Behavioral Intention) and actually use the technology (Use Behaviour). Venkatesh et al describe in the 'Unified Theory of Acceptance and Use of Technology' (UTAUT) that behavioral intention and use behavior are influenced by four core determinants (See Figure 1, (8)). The UTAUT model is based on Innovation and Diffusion Theory (IDT) and social cognitive theories, among others (8). Behavioral intention is the intention to exhibit behavior and usage behavior is the behavior exhibited to use something. The three core determinants that have direct influence on behavioral intention and indirect influence on usage behavior are performance expectancy, effort expectancy, and social influence (8). Facilitating conditions have a direct influence on usage behavior. Performance Expectancy refers to the degree to which an individual expects that using the system will improve their work performance (8). This is linked to the IDT's concept of relative advantage. This is the strongest predictor of intention to use a new technology (8). In addition, there are four moderating factors (gender, age, experience, and voluntariness of use), which influence behavioral intention and behavior. The moderators gender and voluntariness of use are not included in this study because gender is the same for all patients and all respondents participate voluntarily. Age is examined in patients, but firm conclusions are not drawn due to the small numbers. Experience is different for patients and will be included. The group of practitioners is too small to draw conclusions on gender or age.


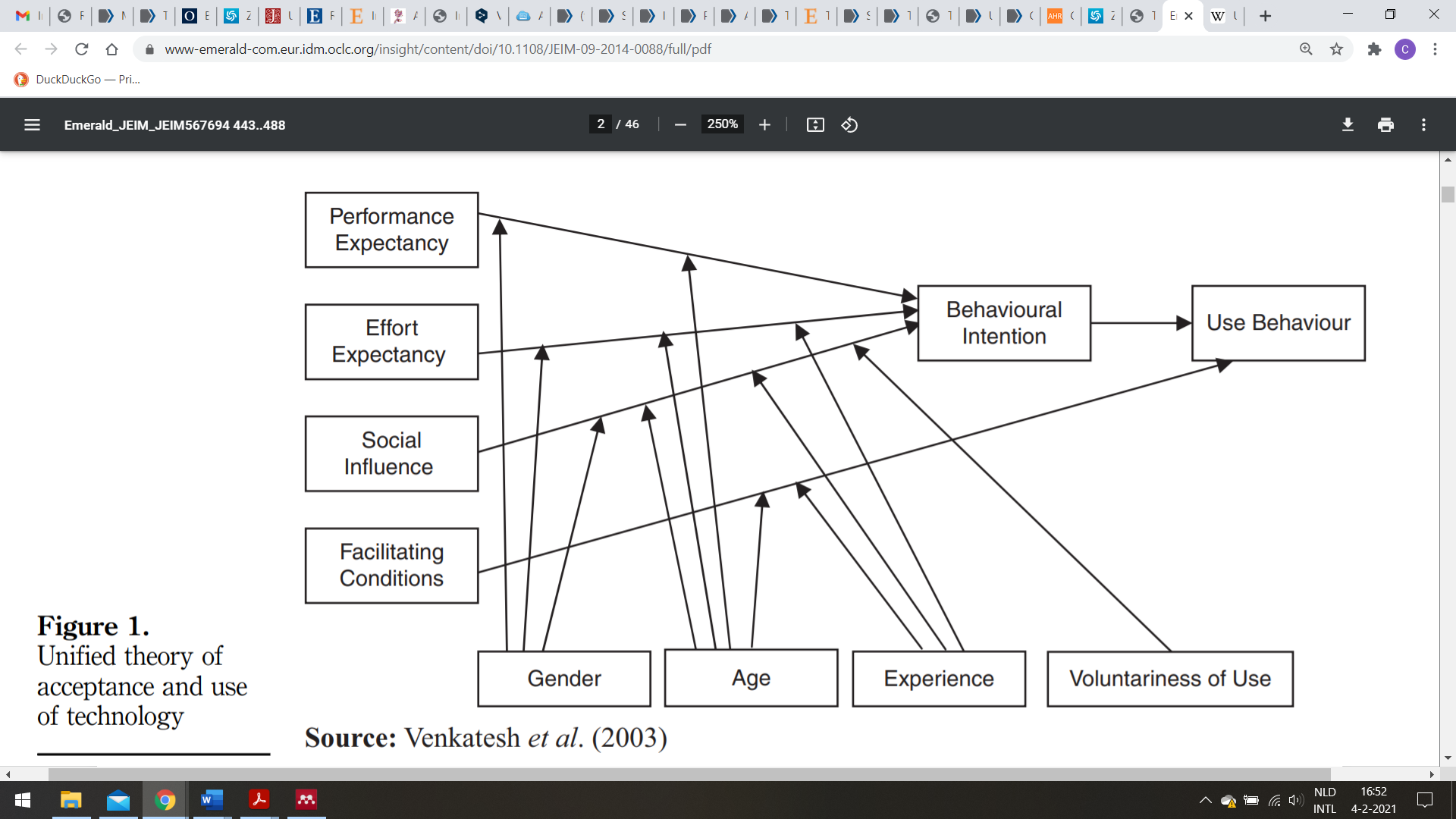


*Figuur 1: Unified Theory of Acceptance and Use of Technology (Venkatesh et al, 2003)*

The model indicates the correlation between the concepts in the model. This research will only look at the correlation and not the significance of that correlation. The model will be used in this qualitative study to explore and describe the performance expectation/relative benefit, (expected) behavioral intention, and usage behavior of the eCoach among patients and healthcare professionals with and without user experience.

### 1.3 NASSS Framework

The Nonadoption, Abandonment, Spread, Scale-up and Sustainability (NASSS) framework was developed to understand and explain why technological innovation programs in healthcare fail (See Figure 2, (4)). The framework brings together classical and implementation theories to form a holistic approach. The NASSS framework was chosen because it aims to understand and/or explain what influences implementation outcomes and can be used to investigate problems of individual adoption (micro-level) and organizational implementation (meso-level) (4).


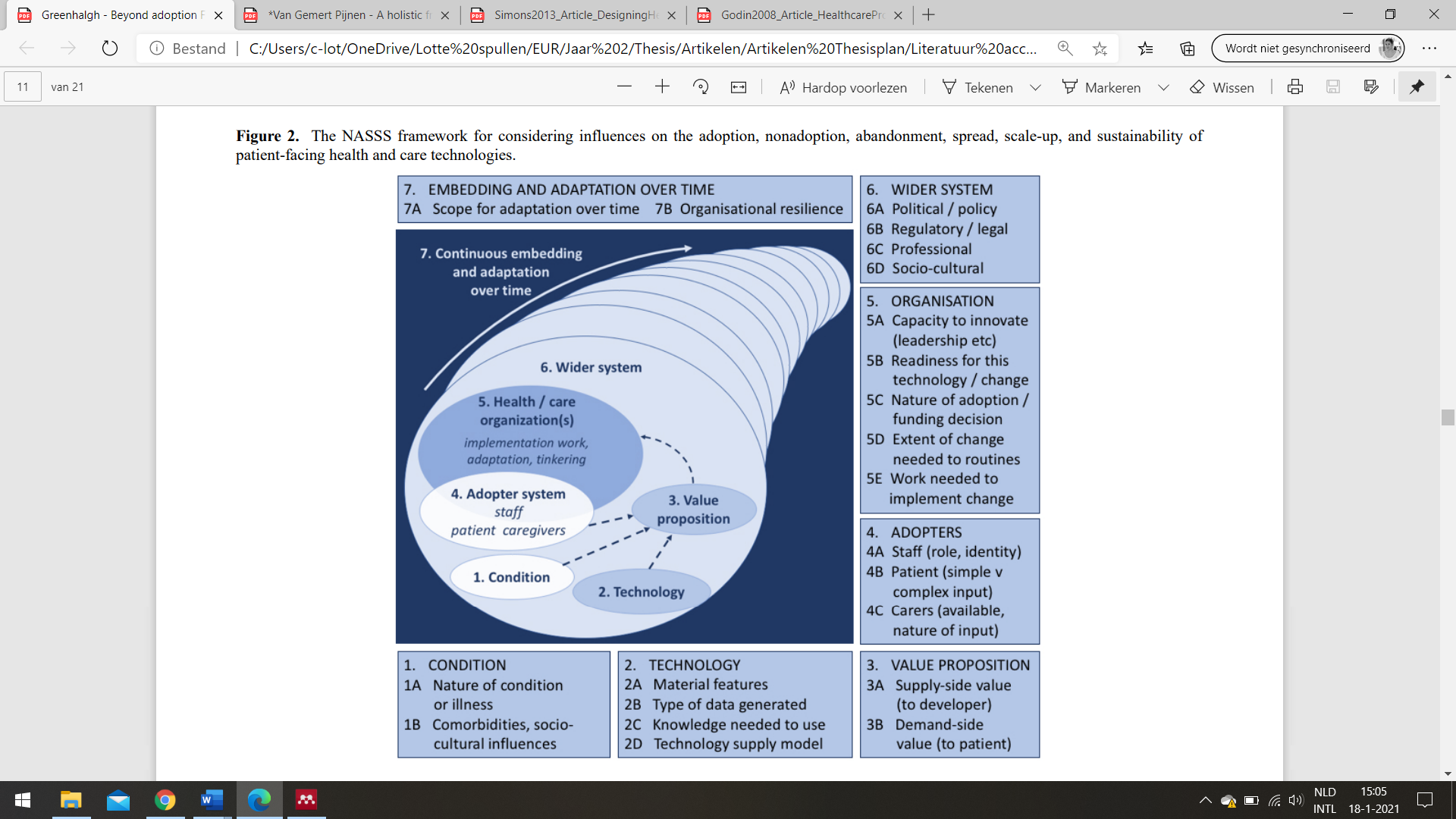


*Figuur 2: Nonadoption, Abandonment, Spread, Scale-up and Sustainability framework (Greenhalgh et al, 2017)*

The NASSS framework consists of seven domains: (1) Condition, (2) Technology, (3) Value proposition, (4) Adopters, (5) Organisation, (6) Wider system, and (7) Embedding and adoption over time. This study focuses on domain 4, but because of the interdependence between the domains, four other domains are included, which is explained below.

The fourth domain concerns adoption and continued use by end-users. Adoption requires that end-users accept the technology, have the intention to use the technology, and use and integrate the technology into their daily lives. The UTAUT model is added in this domain for obtaining and analyzing the data.

The first domain analyzes the condition, related comorbidities, and sociocultural aspects so that the technology aligns with the problem of the condition.

The second domain concerns the material and technical characteristics, the type of knowledge generated, the usability and the problems for sustainable use of the technology.

The third domain "value proposition" concerns whether a technological innovation is worth implementing from the perspective of all stakeholders. This is related to the IDT and affects end-user adoption. The difference in values among stakeholders must be taken into account when developing eHealth technology (4). Therefore, perspectives on the added value of the eCoach from all stakeholders are included.

The fifth domain provides insight into the organizational context. An innovation is more likely to be incorporated into an organization if there is a strong propensity for change, an innovation fits well with existing work routines (innovation system 'fit'), widespread support for the innovation, and systematic evaluations of the consequences of the innovation.

The sixth and seventh domains are outside the scope of this study. The sixth domain concerns the broader (national, institutional and societal) context. The Dutch healthcare system is among the top healthcare systems in the world. This makes the conditions for eHealth implementation in hospitals favorable on a macro-level (3). The seventh domain describes the interaction between the domains and adoption over time. Here, continuous evaluation and improvement of an innovation is necessary for successful implementation is (4).

Per domain a distinction is made in the degree of complexity. Complexity is "a dynamic and constantly evolving set of processes and objects that not only interact with each other, but are also defined by those interactions" (9). A domain can be "simple (unambiguous, predictable, few components), complicated (multiple interacting components), or complex (dynamic, unpredictable, difficult to divide into components)" (10). Based on the complexity of the domains, a prediction can be made as to whether and to what extent an innovation can be successfully implemented. An innovation with multiple simple domains can be easily adopted so implementation will be successful (4). The complexity of the MyRUTIcoach is unknown and will therefore be investigated. Complexity theory has been applied in practical NASSS 'complexity assessment tools' (NASSS-CAT), where the Interview tool can serve as a guide for conducting semi-structured interviews (11). Literature on applying the NASSS-CAT tools are scarce because these tools were developed recently. Lee et al have written a research protocol for evaluating the sustainability of a video-consultation service at the micro-, meso- and macro-level through semistructured interviews based on the NASSS framework (12).

### 1.4 Composed framework

The limitation of the NASSS framework is that it is a generic model that must be adapted to the context of the research. Since this research focuses on micro-level adoption, the UTAUT model is added to further explore domain 4. Therefore, a composite framework is consistent with this research. To our knowledge there is no known literature on combining the NASSS framework and the UTAUT model.

## 2) Relevant domains per end-user or organisational stakeholder

Patient’s interviews focussed on the perception of usability and user friendliness, behavioral intention, adoption (in their daily life) and the added value for them (domains 2, 3 and 4 and determinants of the UTAUT model). Practitioner’s interviews focussed on the condition, the expected acceptance, the user intention, adoption, changes in work routine, and the value added for them (domains 1, 2, 3, and 4). The interviews with organizational stakeholders investigated the technological development, the value proposition, the financial structure, innovation system 'fit', support for the innovation from the organization, change in work routines and experience with implementations of self-management applications (domain 2, 3 and 5). Each domain was analysed to which degree of complexity (simple, complicated or complex) it belongs (4).

## 3) NASSS framework for complexity

The NASSS framework for complexity was used for the complexity analysis of the investigated domains (1).

| NASSS Framework | | | |
| --- | --- | --- | --- |
| Domain/question | Simple | Complicated | Complex |
| **Domain 1: The condition or illness** | | | |
| 1A. What is the nature of the condition or illness? | Well-characterized, well-understood, predictable | Not fully characterized, understood,  or predictable | Poorly characterized, poorly understood, unpredictable, or high risk |
| 1B. What are the relevant socio-cultural factors and comorbidities? | Unlikely to affect care significantly | Must be factored into care plan and  service model | Pose significant challenges to care  planning and service provision |
| **Domain 2: The technology** | | | |
| 2A. What are the key features of the  technology? | Off-the-shelf or already installed,  freestanding, dependable | Not yet developed or fully interoperable; not 100% dependable | Requires close embedding in complex technical systems; significant  dependability issues |
| 2B. What kind of knowledge does the technology bring into play? | Directly and transparently measures [changes in] the condition | Partially and indirectly measures  [changes in] the condition | Link between data generated and  [changes in] the condition is currently  unpredictable or contested |
| 2C. What knowledge and/or support is required to use the technology? | None or a simple set of instructions | Detailed instruction and training  needed, perhaps with ongoing  helpdesk support | Effective use of technology requires  advanced training and/or support to adjust to new identity or organizational role |
| 2D. What is the technology supply model? | Generic, “plug and play,” or  Customizable, off-the-shelf (COTS) solutions requiring minimal customization; easily substitutable if supplier withdraws | COTS solutions requiring significant customization or bespoke solutions; substitution difficult if supplier withdraws | Solutions requiring significant organizational reconfiguration or medium- to large scale-bespoke solutions; highly vulnerable to supplier withdrawal |
| **Domain 3: The value proposition** | | | |
| 3A. What is the developer’s business  case for the technology (supply-side value)? | Clear business case with strong  chance of return on investment | Business case underdeveloped; potential risk to investors | Business case implausible; significant  risk to investors |
| 3B. What is its desirability, efficacy, safety, and cost effectiveness (demand-side value)? | Technology is desirable for patients,  effective, safe, and cost effective | Technology’s desirability, efficacy,  safety, or cost effectiveness is unknown or contested | Significant possibility that technology  is undesirable, unsafe, ineffective,  or unaffordable |
| **Domain 4: The adopter system** | | | |
| 4A. What changes in staff roles, practices, and identities are implied? | None | Existing staff must learn new skills  and/or new staff be appointed | Threat to professional identity, values, or scope of practice; risk of job loss |
| 4B. What is expected of the patient (and/or immediate caregiver)—and is this achievable by, and acceptable to, them? | Nothing | Routine tasks, eg, log on, enter data,  converse | Complex tasks, eg, initiate changes  in therapy, make judgments, organize |
| 4C. What is assumed about the extended network of lay caregivers? | None | Assumes a caregiver will be available  when needed | Assumes a network of caregivers  with ability to coordinate their input |
| **Domain 5: The organization** | | | |
| 5A. What is the organization’s capacity to innovate? | Well-led organization with slack  resources and good managerial relations; risk taking encouraged | Limited slack resources; suboptimal  leadership and managerial relations;  risk taking not encouraged | Severe resource pressures (eg, frozen posts); weak leadership and managerial relations; risk taking may be punished |
| 5B. How ready is the organization for this technology-supported change? | High tension for change, good innovation-system fit, widespread support | Little tension for change; moderate  innovation-system fit; some powerful  opponents | No tension for change; poor innovation-system fit; many opponents, some with wrecking power |
| 5C. How easy will the adoption and funding decision be? | Single organizations with sufficient resources; anticipated cost savings; no new infrastructure or recurrent costs required | Multiple organizations with partnership relationship; cost-benefit balance favorable or neutral; new infrastructure (eg, staff roles, training, kit) can mostly be found from repurposing | Multiple organizations with no formal links and/or conflicting agendas; funding depends on cost savings across system; costs and benefits unclear; new infrastructure conflicts with existing; significant budget implications |
| 5D. What changes will be needed in team interactions and routines? | No new team routines or care  pathways needed | New team routines or care pathways  that align readily with established ones | New team routines or care pathways  that conflict with established ones |
| 5E. What work is involved in implementation and who will do it? | Established shared vision; few simple tasks, uncontested and easily monitored | Some work needed to build shared  vision, engage staff, enact new  practices, and monitor impact | Significant work needed to build  shared vision, engage staff, enact  new practices, and monitor impact |
| **Domain 6: The wider context** | | | |
| 6A. What is the political, economic,  regulatory, professional (eg, medicolegal), and sociocultural context for program rollout? | Financial and regulatory requirements already in place nationally; professional bodies and civil society supportive | Financial and regulatory requirements being negotiated nationally; professional and lay stakeholders not yet committed | Financial and regulatory requirements raise tricky legal or other challenges; professional bodies and lay stakeholders unsupportive or opposed |
| **Domain 7: Embedding and adaptation over time** | | | |
| 7A. How much scope is there for adapting and coevolving the technology and the service over time? | Strong scope for adapting and embedding the technology as local need or context changes | Potential for adapting and coevolving  the technology and service is limited or uncertain | Significant barriers to further  adaptation and/or coevolution of  the technology or service |
| 7B. How resilient is the organization to handling critical events and adapting to unforeseen eventualities? | Sense making, collective reflection, and adaptive action are ongoing and encouraged | Sense making, collective reflection,  and adaptive action are difficult and  viewed as low priority | Sense making, collective reflection,  and adaptive action are discouraged  in a rigid, inflexible implementation  model |

## 4) References

1. Rogers EM. Diffusion of Innovations, Fourth Edition. Elements of Diffusion. 1995.

2. van Gemert-Pijnen L, Kelders SM, Kip H, Sanderman R. eHealth Research, Theory and Development: A Multi-Disciplinary Approach. eHealth Research, Theory and Development. 2018.

3. Faber S, van Geenhuizen M, de Reuver M. eHealth adoption factors in medical hospitals: A focus on the Netherlands. Int J Med Inform. 2017;

4. Greenhalgh T, Wherton J, Papoutsi C, Lynch J, Hughes G, A’Court C, et al. Beyond adoption: A new framework for theorizing and evaluating nonadoption, abandonment, and challenges to the scale-up, spread, and sustainability of health and care technologies. J Med Internet Res. 2017;

5. Nieboer ME, van Hoof J, van Hout AM, Aarts S, Wouters EJM. Professional values, technology and future health care: The view of health care professionals in The Netherlands. Technol Soc. 2014;39.

6. Addotey-Delove M, Scott RE, Mars M. Review of patients’ perspectives of m-health adoption factors in the developing world. Development of a proposed conceptual framework. Informatics Med Unlocked. 2020;

7. van Gemert-Pijnen JEWC, Nijland N, van Limburg M, Ossebaard HC, Kelders SM, Eysenbach G, et al. A holistic framework to improve the uptake and impact of eHealth technologies. Journal of medical Internet research. 2011.

8. Venkatesh V, Morris MG, Davis GB, Davis FD. User acceptance of information technology: Toward a unified view. MIS Q Manag Inf Syst. 2003;

9. Cohn S, Clinch M, Bunn C, Stronge P. Entangled complexity: Why complex interventions are just not complicated enough. J Heal Serv Res Policy. 2013;40-43 (p.42).

10. Greenhalgh T, Wherton J, Papoutsi C, Lynch J, Hughes G, A’Court C, et al. Analysing the role of complexity in explaining the fortunes of technology programmes: Empirical application of the NASSS framework. BMC Med. 2018;

11. Greenhalgh T, Maylor H, Shaw S, Wherton J, Papoutsi C, Betton V, et al. The NASSS-CAT tools for understanding, guiding, monitoring, and researching technology implementation projects in health and social care: Protocol for an evaluation study in real-world settings. JMIR Res Protoc. 2020;

12. Kim SJ, Lee HY. Acute peripheral facial palsy: Recent guidelines and a systematic review of the literature. J Korean Med Sci. 2020;35(30).
