# Supplementary material for: Development and feasibility of a telemedicine tool for patients with recurrent urinary tract infection: myRUTIcoach
Source: Int Urogynecol J. 2023 Sep 27;34(11):2817–25. doi: 10.1007/s00192-023-05634-x (PMC10682280; doi:10.1007/s00192-023-05634-x)
Supplement: Supplementary file 2 — (DOCX 33 kb) [file 192_2023_5634_MOESM2_ESM.docx]

**What is a bladder infection**

The urinary tract consists of the kidneys, ureters, bladder and urethra. A bladder infection is inflammation of the urethra, bladder or both caused by bacteria. A bladder infection is therefore also called a urinary tract infection.

Most bladder infections are caused by the intestinal bacteria Escherichia coli (e. coli). This bacteria enters the urethra and bladder through the poop hole (anus) and the skin. The bacteria can get stuck on the inside of the bladder and cause inflammation.

Women have more frequent bladder infections compared to men. This is because the urethra of women is closer to the anus. This makes it easier for bacteria to reach the urethra. The urethra of women is also shorter: only 3 to 4 cm. More than half of all women get a bladder infection at least once in their lives. About half of those women will develop another bladder infection within a year.

**Risk factors**

There are several factors that increase the risk of a bladder infection.

For women who have not yet gone through menopause, these are mainly sexual activity and the use of condoms, especially condoms with spermicides. Pregnant women are more susceptible to developing a bladder infection. For women who are or have been in the menopause these are urinary incontinence, vaginal dryness due to a reduced amount of female hormones and incomplete urination.

For all women, congenital abnormalities of the urinary tract, a tube in the bladder (catheter), (poorly regulated) diabetes, wiping from the anus to the urethra, drinking less than 2L per day and family members who often have bladder infections are risk factors of getting bladder infections.

**How do I recognize bladder infection**

You can recognize a bladder infection by having to urinate more often than usual. Shortly after urinating, the feeling of having to urinate rises again. In addition, urinating is often painful and often only small amounts or a few drops of urine. The pee may smell strong and look cloudy. Other symptoms of a bladder infection are blood in the urine, pain in the lower abdomen or lower back. The symptoms of a bladder infection are different for everyone. The fact that you do not have one or more symptoms does not mean that you do not have a bladder infection.

A bladder infection can ascend to the kidneys, then there is a kidney infection. Symptoms of a kidney infections include fever, chills, nausea, vomiting, and a continuous aching sensation in the flank.

**I have a bladder infection, what to do?**

If you think you have a bladder infection, there are several thing you can do. In half of the women, a bladder infection will go away on its own. It is important to drink a lot, in order to flush the bacteria out of the bladder. If you suffer from the pain when urinating, you can take paracetamol. If the symptoms do not go away after a few days, you can have your urine checked by the general practitioner. He or she can then decide, after discussing with you, to wait a little longer or to start antibiotics. If you have the aforementioned symptoms that may be consistent with a kidney infection (fever, chills, nausea, vomiting or abdominal pain) you should contact your general practitioner immediately.

**What can I do to prevent another bladder infection.**

You can do several things to minimize the risk of a bladder infection.

- Drink at least 2 liters a day. As a result, you have to go to the toilet more often and bacteria that may be present in the bladder quickly pass out.

- Do not try to hold the pee. Because the urine stays in your bladder longer, bacteria have more chance to grow.

- Take the time to urinate properly. If you feel that you are not urinating properly, it may help to tilt your pelvis. To make this easier, you can place a small step in front of the toilet to put your feet on.

- After going to the toilet it is important that you wipe from the urethra towards the anus. This sweeps bacteria away from the urethra and reduces the chance of them entering your bladder.

- Urinate immediately after intercourse (sex). During sex, bacteria are pushed into the urethra that can cause a bladder infection in the bladder. Urinating after sex removes these bacteria from the bladder.

- If you always have a bladder infection after sexual intercourse, you can take 1 tablet of antibiotics after sexual intercourse. This is something to decide in together with your general practitioner.

- Try to avoid using condoms with spermicides. The use of these condoms increases the risk of developing a bladder infection.

- When your bladder infections are caused by the E. Coli bacteria, taking daily cranberry tablets can help. Cranberries contain a substance that prevents the bacteria from sticking to the wall of your bladder. Other cranberry products such as cranberry juice and fresh cranberries do not work sufficient to prevent the bacteria from sticking to the wall of your bladder.

- When you have been through menopause and the amount of female hormones has decreased, you may suffer from vaginal dryness. You will notice this by a dry or burning sensation in the vagina, pain during intercourse, vaginal itching and a change in your discharge. The acidity of your vagina decreases and the mucous membrane becomes thinner. This makes you more susceptible to getting a bladder infection. If you suffer from these symptoms, a maintenance dose of vaginally administered hormones can cause the acidity to increase again and the mucous membrane to thicken. This gives bacteria less chance to survive and get stuck in your bladder.

- Do not wash your vagina with soap every day. Just like the menopause, this disrupts the acidity of your vagina, making it easier for bacteria to cause inflammation.

- If all the aforementioned measures have no effect, you can use a maintenance dose of antibiotics. This means that you take one tablet of antibiotics every day for a period (usually 3 months). These antibiotics prevent bacteria that get into your bladder from causing an infection.

**An in-depth look at the function and structure of the urinary tract**
The urinary tract consists of the kidneys, ureters, bladder and urethra. In men, the prostate is also part of the urinary tract. The kidneys are located in the back of the abdominal cavity and measure about 12 cm. These are small filters through which the blood flows. The kidneys filter waste products from the blood and ensure that nutrients remain in the body. Depending on the amount of fluid in your body, the kidneys produce more or less urine. If you did not drink sufficient, the urine looks dark yellow to orange. If you drink enough, your urine is light yellow to clear.

The kidneys are connected to the bladder through the ureters. The ureters ensure that the urine ends up in the bladder with undulating movements. The urine is collected in the bladder and when the bladder becomes full, it is put under tension . Then a signal goes through the spinal cord to the brain that you have to urinate (urge). Normally, the bladder is able to hold about 400-600 milliliters of urine. It is important not to delay urination for too long if you feel the urge to urinate. The bladder can then stretch, which may mean that it is no longer able to empty completely.

**In depth description of the treatment of a bladder infection**

If your symptoms do not get better after a few days despite drinking a lot of water and paracetamol or if you have symptoms that are consistent with a kidney infection, your doctor can prescribe antibiotics.

**Antibiotics**

Antibiotics are a form of medication that inhibits the growth of bacteria or kills the bacteria. Antibiotics are not effective for an infection that is not caused by bacteria.

There are several types of antibiotics and it depends on the type of bacteria which antibiotics you receive. For a bladder infection, both the general practitioner and the urologist will usually opt for one of the three standard courses of antibiotics. Depending on the course prescribed, you must take these antibiotics once or for several days.

As with any other medication, antibiotics can cause side effects. The most common side effects of antibiotics are abdominal discomfort, itching and red spots. Itching and red spots can be a side effect, but also a sign that you are allergic to the antibiotics. Other symptoms of an allergy include difficulty breathing, hives or fainting. In the event of these kinds of symptoms, always contact the doctor who prescribed the antibiotics.

It is important that you never decide to stop a course early. If you stop before the course is over, the infection might come back and the bacteria become insensitive (resistant) to the antibiotics. Therefore, always consult with your doctor if you suffer from side effects whether it is safe to stop the antibiotics.

**Resistance**

Resistance means that a bacteria is no longer sensitive to one or more types of antibiotics. The bacteria has then protected itself. It is important to remember that only the bacteria become resistant. You as a person do not become resistant to antibiotics. If a bacteria in your urine is resistant to one or more antibiotics, this will not affect the treatment of an infection elsewhere in your body.

A number of bacteria are already protected against some types of antibiotics. Resistance can also occur when antibiotics are used incorrectly, very often or for a long time. Fortunately, if the bacteria in your urine is resistant to the standard antibiotics, there are other types that your doctor can prescribe you.

Sometimes the bacteria is resistant to all antibiotics that your doctor can prescribe. The hospital has the option of giving you intravenous antibiotics. It used to be necessary for you to be hospitalized during the course of treatment, but nowadays you can also get this treatment at home.
